# A case of neonatal herpes simplex virus type 2 encephalitis with *TLR3* gene mutation and literature review

**DOI:** 10.3389/fneur.2026.1853080

**Published:** 2026-08-05

**Authors:** Xiaojing Li, Yali Chen, Chungui Deng, Dongmei Wang, Jianwu Qiu

**Affiliations:** 1 Department of Neonatology, Yuebei People's Hospital, Guangdong Medical University, Shaoguan, China; 2 Department of Neonatology, Affiliated YueBei People's Hospital of Shantou University Medical College, Shaoguan, China; 3 Guangdong Province Critical Newborn Rescue Center (North Guangdong), Shaoguan, China

**Keywords:** herpes simplex virus type 2, meningoencephalitis, neonate, retinitis, *TLR3* gene

## Abstract

**Background:**

Neonatal herpes simplex virus type 2 (HSV-2) encephalitis frequently manifests with atypical clinical features, which complicates its early identification. Given the challenge of controlling the infant’s seizures, whole exome sequencing was conducted to rule out genetic disorders like early-onset epileptic encephalopathy; this process incidentally revealed a variation in the *TLR3* gene. Host genetic factors, especially the antiviral pathway mediated by TLR3, may influence disease progression.

**Case presentation:**

A 17-day-old female presented with fever and frequent convulsions 15 days after birth. Cranial MRI showed meningoencephalitis, and funduscopy revealed infectious retinopathy. Exome sequencing identified a heterozygous *TLR3* variant (c.338A > C, p. Gln113Pro), and cerebrospinal fluid metagenomic sequencing confirmed HSV-2 infection. Initial cefotaxime-sulbactam plus penicillin was ineffective; subsequent acyclovir and immunoglobulin therapy led to gradual improvement.

**Conclusion:**

In infants with fever and convulsions showing poor response to empirical treatment, cerebrospinal fluid mNGS is strongly recommended for early diagnosis. Further research is needed on the pathogenic role of *TLR3* variants.

## Introduction

Neonatal herpes simplex virus (HSV) infection frequently affects multiple organs and has a poor prognosis. Without prompt treatment, the mortality rate can reach as high as 60%. Even with early acyclovir treatment, severe disabilities may still occur (1). The clinical manifestations of neonatal HSV encephalitis are often atypical, which makes early diagnosis difficult. The gold-standard for pathogen diagnosis is viral PCR in cerebrospinal fluid. However, in atypical or PCR-negative cases, mNGS technology shows unique advantages. Moreover, the host’s genetic background, particularly genes involved in antiviral innate immunity (such as the TLR3 pathway), may influence the clinical course of the disease.

We report a case of neonatal HSV-2 encephalitis confirmed by metagenomics, which primarily presented with fever and convulsions. We also explore the potential significance of the accompanying *TLR3* gene variant, aiming to enhance awareness of the disease and reduce misdiagnosis and missed diagnosis.

## Case presentation

### Medical history and physical examination

The patient, a 17-day-old female infant, was admitted to the hospital due to “fever for 2 days and five convulsions.” She was the first child of the first pregnancy, delivered vaginally at 39 + 1 weeks of gestation, with a birth weight of 3,150 g. She developed a fever 2 days before admission, with a maximum temperature of 39 °C, and was treated with cefotaxime at another hospital. On the day of admission, she had convulsions, manifested as twitching of the left upper limb and both lower limbs, and showed a poor response to phenobarbital treatment. No family history of convulsions was noted. Routine prenatal examinations conducted during early pregnancy at an external hospital showed no significant abnormalities. The mother underwent HSV-IgM antibody testing at an external hospital, which returned a positive result. Subsequent testing at our hospital, however, revealed a nuanced profile: she tested negative for HSV-1 IgM antibodies but positive for HSV-2 IgM antibodies. As the infant was delivered at another hospital and was already 17 days old upon admission, we were unable to complete HSV nucleic acid testing of the mother’s cervical and vaginal secretions.

Upon admission, the physical examination revealed a body temperature of 36.6 °C, a pulse of 145 beats per minute, a respiratory rate of 42 breaths per minute, and a blood pressure of 75/36 mmHg. The infant appeared lethargic, presenting with bilateral conjunctival congestion and yellow discharge. The anterior fontanelle was flat and slightly tense. No obvious abnormalities were detected in the heart and lungs, while muscle tone was increased in both lower limbs. No definite localizing signs were found on the remaining neurological examination.

### Laboratory and imaging tests

Complete Blood Count (CBC), C-reactive protein (CRP), and procalcitonin (PCT) results were normal. On the second day following admission, cerebrospinal fluid (CSF) routine and biochemical analyses revealed the following: a white blood cell count of 24 × 10^6^/L, with lymphocytes predominating at 87.5%; an elevated total protein level of 1.90 g/L; glucose at 2.55 mmol/L; and chloride at 126 mmol/L. Subsequent testing on day five showed a persistent white blood cell count of 25 × 10^6^/L, with lymphocytes comprising 75.0%; a significantly elevated total protein level of 2.50 g/L; glucose decreased to 2.28 mmol/L; and chloride measured at 117 mmol/L. Additionally, the CSF HSV-2 nucleic acid quantitative test yielded a result of 1.13 × 10⁴ Copies/mL. Liver function test results were as follows: total bilirubin 41.10 μmol/L, direct bilirubin 11.10 μmol/L, indirect bilirubin 30.00 μmol/L, alanine aminotransferase 42.00 U/L, aspartate aminotransferase 69.00 U/L. The infant’s complete TORCH screening results, including the test for HSV-2 IgM antibody, all returned negative. Furthermore, both the bilateral eye secretion culture and the specific HSV-2 DNA test also yielded negative findings. Both blood cultures and CSF cultures were negative.

**Figure 1:**
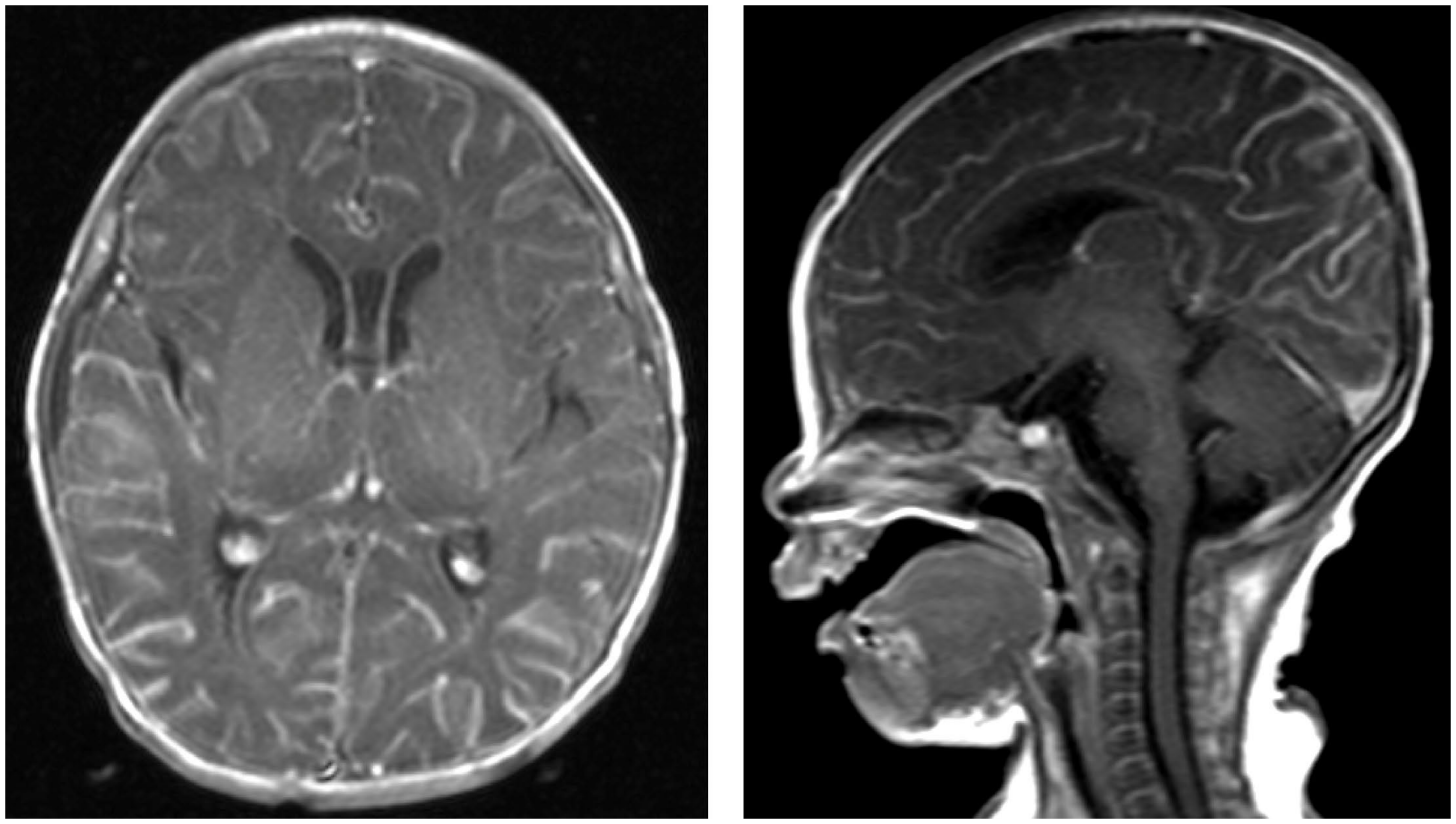
Cranial MRI plain and enhanced scans: Heterogeneous signal intensity of the brain parenchyma in both cerebral hemispheres, with diffuse, pronounced thickening and enhancement of the bilateral meninges.

**Figure 2:**
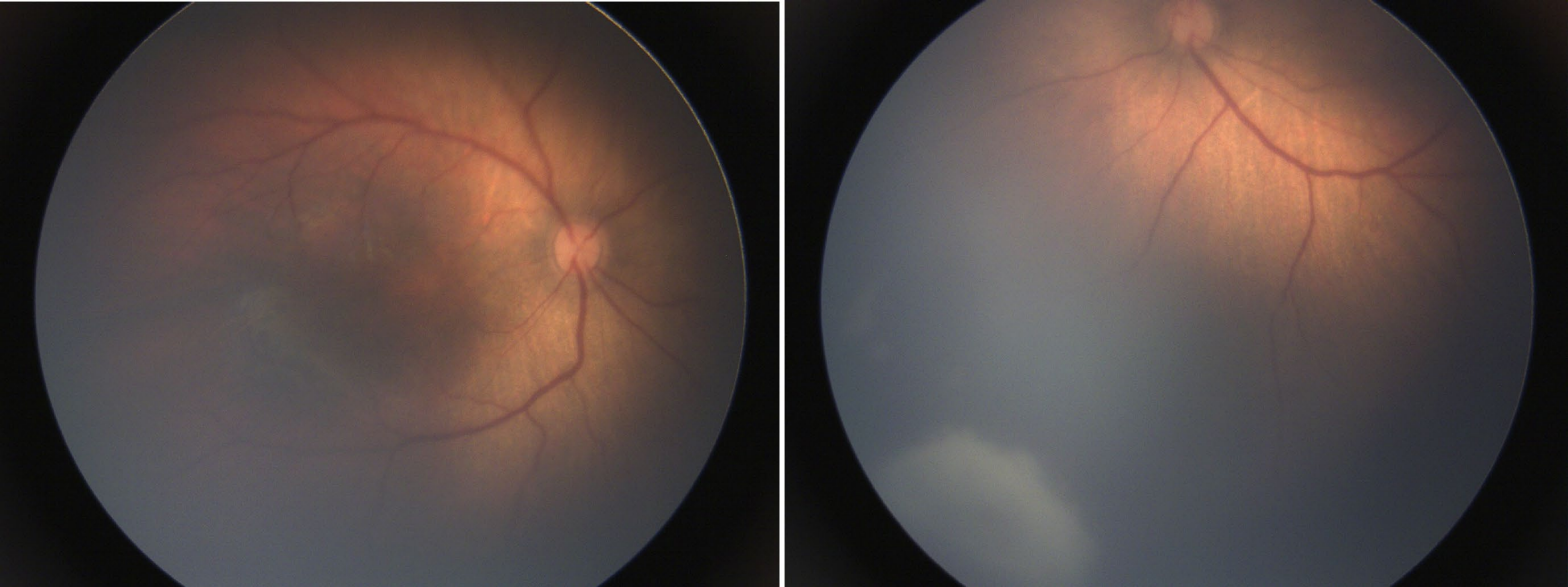
Neonatal fundus examination: Vitreous opacity, with grayish-white patchy exudative lesions observed in the nasal and temporal periphery of the right eye and the nasal periphery of the left eye.

Cranial MRI plain and enhanced scans both revealed extensive meningoencephalitis (Figure 1). The brain parenchyma within bilateral cerebral hemispheres exhibited a heterogeneous signal pattern, showing extensive hyperintensity on T2W images and hypointensity on T1W sequences, alongside multiple scattered, slightly hyperintense signal changes on DWI. The enhanced scan demonstrated diffuse and markedly thickened meninges with pronounced enhancement across both cerebral hemispheres. Cerebellum and brainstem normal in morphology and signal. Suprasellar and cavernous sinus structures unremarkable. All ventricles, cerebral sulci, and cisterns normal in morphology, size, and shape. Electroencephalogram (EEG) showed low-amplitude brain waves. Cranial ultrasound suggested widening of the lateral ventricles. Fundus examination revealed infectious retinopathy in both eyes (Figure 2).

### Diagnosis

Given the infant’s alarming clinical deterioration and the failure of routine testing to pinpoint a cause, the possibility of early-onset epileptic encephalopathy could not be ruled out. Consequently, we proceeded with whole exome sequencing (WES). To identify the pathogen with utmost urgency, we also employed metagenomic next-generation sequencing (mNGS) technology, seeking a more precise and definitive etiological diagnosis. On the fifth day of hospitalization, mNGS of cerebrospinal fluid (CSF) detected HSV-2-specific sequences (1,017 reads). CSF HSV-2 nucleic acid testing yielded a positive result.

The infant presented with fever and frequent seizures. Initial bloodwork, including CBC, CRP, and PCT, returned normal results. However, analysis of the cerebrospinal fluid revealed a mild elevation in white blood cells and protein, with a notable increase in lymphocytes. Imaging studies further clarified the picture: cranial MRI indicated meningoencephalitis, while a fundus examination showed bilateral infectious retinopathy. The HSV-2 virus was identified in the cerebrospinal fluid via mNGS, a finding corroborated by a positive HSV-2 nucleic acid test. This result was further supported by the mother’s positive serum HSV-2 antibody test. This finding was supported by the mother’s positive serum HSV-2 antibody test. The collective evidence led to a diagnosis of neonatal HSV-2 encephalitis.

### Treatment process

After admission, given the infant’s critical condition and the inability to rule out Group B Streptococcus (GBS) infection, we promptly initiated empirical therapy with cefoperazone-sulbactam in combination with penicillin. In addition, phenobarbital was administered for antiepileptic therapy. Mannitol was administered to combat the symptoms of elevated intracranial pressure, notably the infant’s flat and distinctly tense anterior fontanelle. However, the infant still had fever and convulsions. Consequently, midazolam and sodium valproate were added for combined anticonvulsant therapy.

Following the confirmation of HSV-2 encephalitis, antibiotics were promptly ceased, and intravenous acyclovir (20 mg/kg/dose, q8h) was initiated for a 21-day antiviral course, supplemented with intravenous immunoglobulin (700 mg/kg/day × 3 days). Subsequently, the infant’s fever gradually subsided and seizure frequency diminished. After 1 month of hospitalization, the infant was discharged in improved condition.

### Genetic sequencing findings

**Figure 3:**
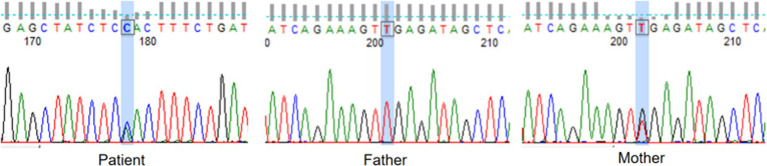
Family Sanger sequencing validation image: *TLR3* gene c.338A > C, the variant was maternally inherited.

WES of the family identified a heterozygous variant in the *TLR3* gene, c.338A > C (p. Gln113Pro), which was inherited from the mother (Figure 3). The pathogenicity of this variant is unknown.

### Follow-up and outcome

**Figure 4:**
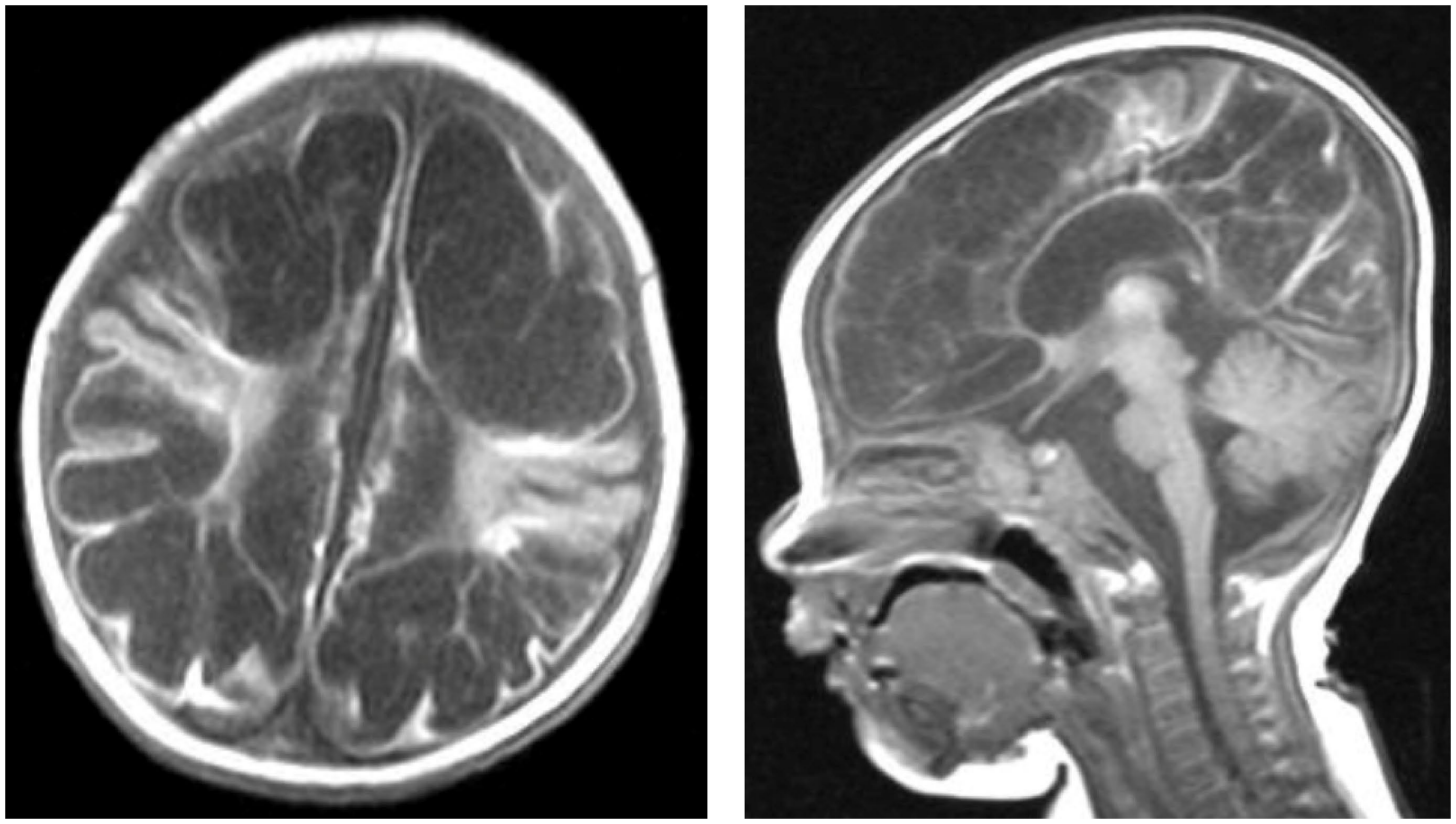
Follow-up cranial MRI plain scan: Thinning of the cerebral cortex in both hemispheres, widening and deepening of the sulci and cisterns, extensive encephalomalacia in both cerebral hemispheres, and dilatation of the supratentorial ventricles.

The infant continued oral acyclovir for 1 month after discharge. A follow-up cranial MRI at 4 months of age showed extensive encephalomalacia in both cerebral hemispheres (Figure 4). Compared with previous MR images: the cortex of bilateral cerebral hemispheres was thinned, with widened and deepened cerebral sulci, fissures, and cisterns; brain parenchyma showed extensive hyperintensity on T2W, hypointensity on T1W, and hypointensity on T2 FLAIR and diffusion; supratentorial ventricles were dilated. These findings were considered sequelae changes of meningoencephalitis. After more than 5 months of follow-up, the infant still had intermittent convulsions and required long-term oral sodium valproate for antiepileptic treatment. The long-term neurological impact awaits further assessment.

## Literature review

### Methods

To gain a deeper understanding of the disease, a comprehensive literature search was conducted using the terms “herpes simplex virus-2 encephalitis.” The following filters were applied: Newborn: birth – 1 month. The search was carried out in databases including PubMed, Web of Science, and Chinese databases (China National Knowledge Infrastructure, Wanfang Data Knowledge Service Platform, and VIP Information Journal Database) from 2000 to 2025.

A total of 38 cases of neonatal HSV-2 encephalitis were included, encompassing 26 published studies (2–27). These cases were analyzed from multiple dimensions, including age of onset, clinical manifestations, pharmacological treatment, and prognosis. Specific data are presented in Supplementary Table S1.

### Results

Based on the geographic information provided in the literature, the distribution unfolds as follows: the United States leads with 15 cases, constituting 39.5% of the total, followed by China with 11 cases, representing 28.9%. Greece, Canada, Japan, and South Korea each reported 2 cases, accounting for 5.3%, respectively. Sweden, the United Kingdom, Italy, and Bulgaria each contributed 1 case, making up 2.6% each. Full-term infants represented 71.4% (15/21) of the cohort, while preterm births comprised 28.6% (6/21). Male infants accounted for 48.1% (13/27) of the group, with females making up the remaining 51.9% (14/27). Vaginal delivery was the mode for 86.4% (19/22) of births, whereas cesarean section accounted for 13.6% (3/22). The median age of onset was 15.5 (11.0, 21.0) days.

Neurological manifestations predominated as the primary symptoms, affecting 78.4% (29/37) of cases. These specifically encompassed convulsions, heightened or diminished limb muscle tone, somnolence, impaired responsiveness, and profound coma. Fever ranked as the second most frequent symptom, occurring in 45.9% (17/37). Respiratory symptoms—including tachypnea, apnea, respiratory distress, and labored breathing—manifested in 21.6% (8/37). Acute retinitis was also documented in 10.8% (4/37). The overwhelming majority (91.9%, 34/37) were late-onset infections, emerging beyond 3 days. Cranial imaging revealed abnormalities in 91.4% (32/35) of patients; findings varied from focal lesions to diffuse, extensive damage, often progressing to cerebral softening and atrophy. Abnormal electroencephalograms (EEGs) were detected in 86.7% (13/15) of cases, primarily showing mild nonspecific abnormalities or diffuse, severe brain dysfunction, frequently accompanied by epileptiform discharges. The median cerebrospinal fluid (CSF) white blood cell (WBC) count was 153.50 (48.50, 250.25)/μL, and diminished CSF glucose (GLU) levels were noted in 75.0% (12/16). In terms of diagnosis, PCR testing confirmed 78.4% (29/37) of cases, whereas only 8.1% (3/37) relied on the mNGS method. Regarding treatment, commonly administered agents included acyclovir and ganciclovir. Overall prognosis was markedly poor, with 23 patients exhibiting impaired later development, including 6 documented fatalities.

## Discussion

Neonatal HSV-2 encephalitis often progresses stealthily, manifests with nonspecific symptoms, and poses significant challenges for early diagnosis. When treatment is delayed, both the mortality rate and the incidence of long-term neurological sequelae become severely elevated.

### Biology of HSV infection

The HSV is an enveloped, double-stranded DNA virus categorized into two types. Type 1 primarily causes infections of the oral cavity and pharynx, whereas type 2 mainly affects the genital tract. Notably, HSV type 2 is a significant pathogen in neonatal infections, often leading to more severe disease and higher mortality rates. The routes of neonatal HSV infection include placental transmission (about 5%), transmission through the birth canal (about 85%), and postnatal infection (about 10%) (28). The incidence of neonatal HSV infection is approximately 10 cases per 100,000 live births.

### Clinical characteristic analysis

Clinically, it manifests in three distinct forms: localized skin, eye, and mouth infections (SEM), central nervous system infections, and disseminated HSV infections. The mortality rate for neonates with disseminated HSV infection lingers at 29%, while over 65% of survivors with central nervous system involvement suffer from lasting neurological sequelae (29). After HSV infection, infants mainly present with fever and poor responsiveness, and the condition progresses rapidly, involving the respiratory system, nervous system, liver, and other organs. They are also prone to shock and disseminated intravascular coagulation. Cranial imaging may provide evidence of brain damage (2). Among these, HSV-2 stands as a recognized cause of neonatal encephalitis. Residing within the genital tract, the virus can be transmitted to neonates through intrauterine ascending infection or during delivery via an infected maternal birth canal (3). A minority of cases may also result from placental transmission or contact transmission involving hospital staff or family members. Neonatal HSV-2 encephalitis represents a form of central nervous system infection—a rare yet devastating disease characterized by high mortality and severe neurological sequelae. Case reports of this condition remain scarce in both domestic and international literature.

In this case, the infant developed symptoms 15 days after birth, initially presenting with fever and convulsions. Notably absent were skin or oral lesions, multi-organ failure, or elevated early inflammatory markers, effectively ruling out SEM or disseminated HSV infection. Central nervous system infection emerged as the more probable diagnosis. This study found that neonatal HSV-2 encephalitis is more common in full-term infants and those delivered vaginally. Seizures, changes in muscle tone, and other neurological manifestations, as well as fever, are the most common clinical symptoms of this disease. The vast majority of patients (91.7%) exhibited abnormal cranial imaging findings, typically presenting as focal to diffuse extensive damage that often advanced to encephalomalacia and atrophy; in this particular instance, the presentation involved diffuse extensive damage, which subsequently evolved into extensive encephalomalacia accompanied by ventricular dilation. Concurrently, abnormal EEG results were observed in most cases (86.7%), while decreased cerebrospinal fluid glucose levels were identified in 75.0% of patients.

Notably, this infant presented with concurrent ocular lesions alongside HSV-2 encephalitis. HSV-2 ocular involvement can present as acute retinal necrosis, optic neuritis, or keratitis, carrying an especially pronounced risk of blindness in neonates and immunocompromised individuals. The overwhelming majority of neonatal HSV retinitis cases coincide with central nervous system infection. Approximately 20% of HSV infections may involve conjunctivitis, keratitis, or dermatitis. When HSV encephalitis occurs, the incidence of ocular infection surges significantly to 60%, with 20–25% of these cases potentially advancing to retinitis. Nearly all instances of neonatal HSV retinitis stem from HSV-2 (30). Direct HSV-2 viral invasion or immune-mediated demyelination reactions can provoke autoimmune responses within the central nervous system, culminating in bilateral optic neuritis (31). HSV-2 can establish latency within the trigeminal ganglion, later traveling via axons to the retina and inciting necrotizing retinitis (32). However, this study observed acute retinitis in only 10.5% of infants, a lower proportion than previously documented. We hypothesize this may relate to more timely interventions over the past two decades and a resultant reduction in ocular infection incidence. This conclusion warrants additional investigation and validation with a larger cohort.

### Application of diagnostic technologies

However, due to vague and non-specific clinical manifestations, negative serum HSV-2 IgM results, and inconclusive routine cerebrospinal fluid examination findings, early diagnosis posed a significant challenge. Currently, the diagnosis of HSV encephalitis primarily relies on cerebrospinal fluid PCR (33) and mNGS (34), while imaging serves as crucial supportive evidence and serology holds only auxiliary value. mNGS proves particularly valuable in confirming challenging cases of HSV encephalitis; given the complexity of this presentation, we proceeded with cerebrospinal fluid mNGS testing.

The pivotal diagnostic breakthrough arrived later: mNGS of the cerebrospinal fluid detected HSV-2 virus, definitively identifying it as the primary pathogen responsible for the infant’s encephalitis. This mNGS technology—leveraging next-generation sequencing to comprehensively analyze microbial and host genetic material (DNA and RNA) within patient samples for pathogen identification—is rapidly transitioning from research into clinical laboratories (35). Clinically, cerebrospinal fluid mNGS proves invaluable for diagnosing herpes simplex virus-2 encephalitis and guiding effective treatment, yielding favorable outcomes (36). The mNGS test, when conducted early in the disease course, can also uncover cases overlooked by PCR diagnosis (34). For challenging and severe neonatal central nervous system infections, mNGS emerges as an unbiased detection tool of significant clinical utility, serving as both a vital complement and a potentially superior alternative to conventional diagnostic methods.

While mNGS boasts outstanding advantages, it typically requires 3 days to deliver a report, whereas PCR can provide results within a single day. For cases with a high suspicion of HSV infection, PCR testing may be prioritized, and empirical acyclovir treatment should be initiated as early as possible. In this instance, due to the infant’s critical condition, the limited volume of available cerebrospinal fluid sample, and negative serum HSV antibody test results—which failed to confirm HSV infection—cerebrospinal fluid HSV PCR testing was not performed simultaneously or given priority. We also engaged in thorough discussions with the family regarding the potential use of acyclovir; the family expressed a preference to await the mNGS test results to avoid unnecessary adverse reactions from empirical acyclovir use, and thus acyclovir treatment was not initiated in a timely manner. For neonates with suspected HSV encephalitis, mNGS can serve as an ultimate diagnostic tool, while rapid HSV PCR testing and immediate empirical antiviral therapy should be equally emphasized.

### Maternal-to-child transmission mechanisms

In this case, the infant’s mother exhibited no symptoms of genital HSV infection yet tested positive for HSV-IgM antibodies. Consequently, the vaginal delivery emerged as a critical factor for HSV transmission. HSV-2, residing within the genital tract, may be acquired by neonates through intrauterine ascending infection, postnatal exposure, or direct contact with the infected maternal birth canal. Genital HSV infections during pregnancy are predominantly recurrent. However, most neonatal HSV infections stem from the mother acquiring a novel HSV serotype during gestation. Approximately two-thirds of women contracting new infections during pregnancy display no genital herpes symptoms, possess no history of the condition, nor do their partners report such a history (3).

### *TLR3* variant and HSV-2 encephalitis

The HSV is a DNA virus rather than an RNA virus, with a linear double-stranded DNA genome. However, during HSV infection, viral transcription produces a large number of overlapping transcripts, which subsequently form double-stranded RNA (dsRNA) structures. The *TLR3* gene encodes the endosomal transmembrane receptor Toll-like receptor 3, which plays a key role in the body’s antiviral defense by specifically recognizing dsRNA produced during viral infection. Congenital *TLR3* immunodeficiency has been firmly established as a critical factor heightening susceptibility to life-threatening HSV-1 encephalitis in children (37); however, any potential link to increased susceptibility to HSV-2 encephalitis remains unreported. Mutations disrupting five critical genes within the TLR3 signaling pathway—*TLR3*, *UNC93B1*, *TRIF*, *TRAF3*, and *TBK1*, which encode key downstream components—have been identified in pediatric HSV encephalitis (HSE) patients. These genetic alterations impair interferon (IFN)-*α*/*β* production within the central nervous system (CNS), suggesting that compromised intrinsic TLR3–IFN-α/β immunity underlies HSE pathogenesis in children (38). *TLR3* gene variants significantly increase vulnerability to severe viral infections (37). In the present case, the infant harbored a *TLR3* gene variant, c.338A > C (p. Gln113Pro). This substitution replaces the neutral polar amino acid glutamine with the neutral nonpolar amino acid proline in the TLR3 peptide chain. Notably, this variant exists in population databases (rs559783546, gnomAD frequency 0.04%). While multiple computational algorithms (REVEL 0.31, SIFT4G 0.316, PolyPhen2_HDIV 0.004, etc.) predict this variant site as benign or of uncertain significance, and crucially, it has not been reported in patients with TLR3-related disorders, whether it elevates the risk of herpetic encephalitis demands further functional validation.

## Conclusion

Neonatal HSV-2 encephalitis has an insidious onset, lacks specific clinical manifestations, and is difficult to diagnose early, with high mortality rates and long-term neurological sequelae. When neonates present with unexplained fever and seizures and show poor response to conventional anti-infective treatment, this disease should be highly suspected. Cerebrospinal fluid mNGS technology can rapidly identify the pathogen, providing critical evidence for early diagnosis and targeted treatment, and has significant application value in the diagnosis of difficult neonatal central nervous system infections. HSV-2 encephalitis is often associated with ocular involvement, and ocular screening should be emphasized in clinical diagnosis and treatment to avoid missed diagnosis of related lesions. The *TLR3* gene heterozygous variation c.338A > C (p. Gln113Pro) detected in this case is a variant of uncertain significance. Based on existing research findings, it is speculated that this variation may increase the susceptibility of the infant to herpes simplex virus central nervous system infection by affecting the TLR3-mediated antiviral immune pathway, and its specific pathogenic effects still require further functional studies for validation. More cases need to be accumulated in the future to deeply explore the association between gene variations and the onset of neonatal HSV-2 encephalitis, providing evidence for disease risk screening, pathogenesis elucidation, and individualized diagnosis and treatment.

## Data Availability

The original contributions presented in the study are included in the article/ Supplementary material, further inquiries can be directed to the corresponding author.
